# Loss of RMI2 Increases Genome Instability and Causes a Bloom-Like Syndrome

**DOI:** 10.1371/journal.pgen.1006483

**Published:** 2016-12-15

**Authors:** Damien F. Hudson, David J. Amor, Amber Boys, Kathy Butler, Lorna Williams, Tao Zhang, Paul Kalitsis

**Affiliations:** 1 Murdoch Childrens Research Institute, Royal Children’s Hospital, Melbourne, Parkville, Victoria, Australia; 2 Department of Paediatrics, University of Melbourne, Parkville, Victoria, Australia; 3 Cytogenetics Laboratory, Victorian Clinical Genetics Services, Murdoch Childrens Research Institute, Royal Children’s Hospital, Melbourne, Parkville, Victoria, Australia; University of Washington School of Medicine, UNITED STATES

## Abstract

Bloom syndrome is a recessive human genetic disorder with features of genome instability, growth deficiency and predisposition to cancer. The only known causative gene is the BLM helicase that is a member of a protein complex along with topoisomerase III alpha, RMI1 and 2, which maintains replication fork stability and dissolves double Holliday junctions to prevent genome instability. Here we report the identification of a second gene, *RMI2*, that is deleted in affected siblings with Bloom-like features. Cells from homozygous individuals exhibit elevated rates of sister chromatid exchange, anaphase DNA bridges and micronuclei. Similar genome and chromosome instability phenotypes are observed in independently derived *RMI2* knockout cells. In both patient and knockout cell lines reduced localisation of BLM to ultra fine DNA bridges and FANCD2 at foci linking bridges are observed. Overall, loss of RMI2 produces a partially active BLM complex with mild features of Bloom syndrome.

## Author Summary

Cells contain specific protein complexes that are needed to correct errors during the replication and segregation of DNA. Impairment in the activity of these proteins can be detrimental to the viability of the cell and organism development. Bloom syndrome is an example of a genome instability disorder where cells cannot efficiently untangle DNA after replication. The only gene that is known to cause Bloom syndrome is the BLM helicase. In this article, we describe two affected individuals with Bloom-like features with a homozygous deletion of the RMI2 gene. The RMI2 protein has previously been shown to form a complex with BLM, topoisomerase III alpha and RMI1. Deletion of RMI2 in patient and unrelated cell lines show hyper-recombination and chromosome entanglements during cell division. Furthermore, we show that the BLM and FANCD2 proteins are diminished in the binding of DNA bridges that need to be dissolved during the late stages of cell division. Therefore, loss of RMI2 produces a milder Bloom phenotype and impairs the full activity of the BLM complex.

## Introduction

Bloom syndrome (BS) is a very rare genetic disorder with features of significant growth deficiency, hypo- and hyperpigmented skin, sun-sensitive facial skin lesions, cancer predisposition in early life and male infertility [1,2]. Early cytogenetic experiments revealed clues about the underlying mechanism with patient chromosomes exhibiting hyper-recombination and genome instability [3]. The only known gene, BLM, associated with BS was identified in 1995 [4]. The gene encodes for the BLM protein that is a member of the RecQ DNA helicase family of proteins. RecQ helicases are essential for genome maintenance and are conserved across evolution.

Protein interaction studies have shown that the BLM protein is a member of a four-subunit complex that includes topoisomerase III alpha (TOP3A) [5,6] and RecQ-mediated genome instability proteins 1 [7–9] and 2 [10,11] (RMI1 & 2), collectively known as the BTR complex. The BTR promotes the dissolution of double Holliday junctions that can be formed during DNA replication into non-crossover products in a two-step process: 1) by pushing the Holliday junctions together by the helicase activity of BLM, and 2) the dissolution of hemi-catenated DNA by the cleavage and joining activities of TOP3A [12]. Crossover events between homologs in somatic cells can be detrimental to a cell’s survival as they lead to loss of heterozygosity (LOH) [13,14,15]. Notably LOH is elevated in BLM deficient cells [16]. Moreover, unresolved recombination intermediates that persist into mitosis lead to bridging and are a source of genomic instability [17]. Structure and function studies have shown that RMI1 and 2 form a heterodimer that is important in stabilising the BTR [18,19]. The BTR complex has been proposed to localise to stalled replication forks via interactions with Fanconi anaemia (FANC) subunits and Replication Protein A [20]. This super-complex is also known as BRAFT. Similar to BS, Fanconi anemia patients exhibit growth deficiencies, chromosomal breaks, heightened genomic instability and cancer predisposition [21]. Further evidence to support this connection is through structural analyses with a FANCM peptide and the RMI1-RMI2 heterodimer [22].

The FANC core complex consists of eight subunits that promote the monoubiquitination of FANCD2 and FANCI in response to sites of DNA damage where replication forks are obstructed [23,24]. FANCD2 acts at stalled replication forks to remove interstrand cross-links (ICLs) and additionally regulates homologous recombination proteins including BRCA2/FANCD1 [25–27]. BLM is known to cooperate with FANCD2 during S phase to restart stalled replication forks while also suppressing the firing of new replication origins; an activity that is independent of FANCI [28]. During mitosis, FANCD2 and FANCI subunits frequently appear at the sister chromatid anchor sites that link DAPI-negative chromatin threads also known as ultra fine bridges (UFBs) and also occasionally along the UFBs during anaphase [29,30]. FANCI/D2 sister foci in mitosis appear at chromosome arms and not centromeres and their localisation corresponds to fragile sites in the genome [29]. The foci that link UFBs during chromosome segregation imply a tethering or loading function for proteins that coat UFBs such as BLM and PICH [31,32], but this is yet to shown.

BLM, TOP3A and RMI1 are highly conserved in most eukaryotes but RMI2 is absent in some lineages including invertebrates and yeasts, suggesting that it is needed in organisms with higher genome complexity [11]. Further evidence to support RMI2’s functional role in higher eukaryotes was shown in chicken DT40 RMI2 null cells which display elevated levels of sister chromatid exchanges (SCEs). At a cellular level, whether RMI2 is required during mitosis and at an organism level, its role during development and disease predisposition are all outstanding questions. Here, we show a homozygous deletion of *RMI2* in two siblings with milder clinical features of Bloom syndrome. We have additionally mutated *RMI2* using CRISPR-Cas9 gene-editing to further confirm the hyper-recombination and mitotic defect phenotypes observed in the patients’ cells.

## Results

### Homozygous deletion of *RMI2* in a family with Bloom-like features

The two affected siblings are the only children of first cousin parents of Pakistani descent. Their mother had one previous miscarriage but there was no other relevant family history. The clinical features of both siblings are summarised in Table 1.

**Table 1:** Clinical features of RMI2-deleted siblings.

Feature	Bloom syndrome	Sibling 1	Sibling 2
Prenatal growth deficiency	+	-	+
Postnatal growth deficiency	+	-	+
Decreased subcutaneous fat	+	-	-
Photosensitivity	+	-	-
Feeding difficulties	+	-	-
Gastro-esophageal reflux	+	-	+
Recurrent infections	+	-	-
Learning difficulties	+ (variable)	-	-
Cancer onset in early adulthood	+	None at age 6	None at age 4
Diabetes	+	Not at age 6	Not at age 4
Café-au-lait macules	+	++	++
Increased sister chromatid exchange	+	+	+

**Fig 1:**
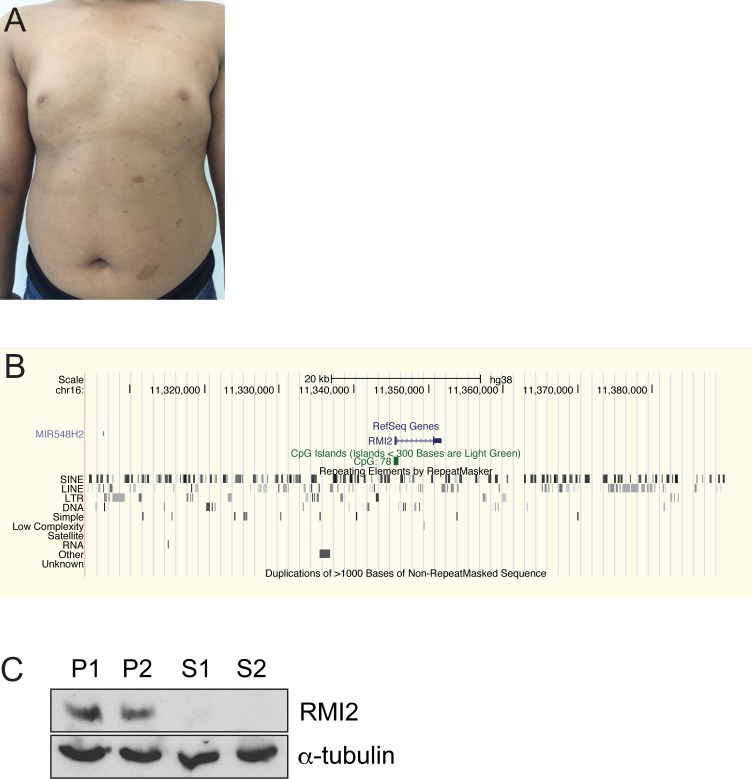
Bloom-like features in two siblings with an RMI2 gene deletion. (A) Sibling S1 shows typical café-au-lait macules on torso that are a common feature of Bloom syndrome. (B) UCSC Genome Browser view of the homozygous deleted region that spans the entire RMI2 gene. (C) Immunoblot confirms complete loss of the RMI2 protein in each sib, S1 and S2. Heterozygous parents, P1 and P2, show the presence of RMI2. Anti-α-tubulin was used a protein loading control.

Sibling 1 (S1 Fig, Fig 1A), a male, was born by caesarean section at 36 weeks gestation. Birth weight was 2.7 kg (50th centile) but other growth parameters were not recorded. He was noted to have large numbers of café-au-lait macules in the first year of life. The café-au-lait macules were mostly <1cm in diameter, but several were >5cm (Fig 1A). There were no other features of neurofibromatosis type 1. There were also several depigmented macules. He was otherwise healthy with normal growth and development, and was an average student at school. At age six years his height was 118 cm (50th-75th centile), weight 28.8 kg (75th-90th centile) and head circumference 50.0 cm (50th centile). There was no cutaneous photosensitivity, reduction in subcutaneous fat, or feeding difficulties or recurrent infections. Neurological, cardiac, respiratory and abdominal examinations were normal and he did not have the characteristic facial appearance of Bloom syndrome. Full blood examination, electrolytes, blood glucose, liver function, and immune function were normal but alpha fetoprotein was mildly elevated.

Sibling 2 (S2), a female, was born at 37 weeks gestation following a pregnancy that was complicated by slow growth in the 3rd trimester. She was delivered by caesarean section and birth weight was 2.2 kg (<10th centile). Other growth parameters were not recorded. There were no neonatal complications. Gastro-esophageal reflux was diagnosed at age one month and was treated medically until age ten months. Multiple café-au-lait macules were noted in the first year of life with a similar pattern to her brother. Her growth was mildly delayed and she was microcephalic: at age four years her height was 95.0 cm (5th centile), weight 15.0 kg (25th centile) and head circumference 45.5 cm (1 cm below 2nd centile). S2 was otherwise healthy and hearing, vision, voice and development were normal. There was no cutaneous photosensitivity or reduction in subcutaneous fat and there was no history of recurrent infections. Neurological, cardiac, respiratory and abdominal examinations were normal and she did not have the characteristic facial appearance of Bloom syndrome. Full blood examination, electrolytes, blood glucose, liver function, and immune function were normal but alpha fetoprotein was mildly elevated.

### Deletion characteristics and mechanism

The mild growth abnormalities of S2 and the presence of café-au-lait macules in both S1 and S2 suggested an underlying genetic defect and a chromosome microarray was requested for the affected family members. The microarray analysis in both siblings demonstrated long continuous stretches of homozygosity consistent with the parents being first cousins. A homozygous deletion was detected comprising 80 kb at chromosome band 16p13.13, resulting in the deletion of the entire *RMI2* gene and the micro RNA gene, *MIR548H2* (Fig 1B and S1 Fig). Both parents were heterozygous for the same deletion. Of note, *BLM* was not within a region of homozygosity.

To identify the exact breakpoint region, oligonucleotides were designed adjacent to the closest positive array probe at each breakpoint. Long-range PCR produced a band of approximately 6 kb for both affected children, whereas an unrelated control displayed no fragment. Sequencing of the cloned PCR product revealed a non-allelic recombination event between two Alu repeat elements, without any loss or gain of Alu sequences (S2 Fig). The deletion therefore covers a region of 84,871 bp located at chr16: 11,304,701–11,389,571 (hg38) (Fig 1C). Aside from *RMI2*, the deleted region contained no other coding genes.

The two Alu repeat elements share an overall sequence identity of 80% spanning 308 bp. Interestingly, a continuous stretch of 38 bp showing 100% identity between the repeats crossed the breakpoints. The deleted region does not span any copy number variable region and contains no known segmental duplication of >1000 bp as displayed on the UCSC Genome Browser.

### RMI2 suppresses sister chromatid exchange events and mitotic defects in patient cells

Routine G-banding analysis on lymphocytes showed no gross chromosomal rearrangements. S1 was 46,XY in 15 metaphase cells, and S2 was 46,XX in 15 metaphase cells. Solid staining for chromosomal breaks in 100 metaphase cells revealed a higher rate in the affected siblings. 15 and 5 chromosome or chromatid breaks were identified in S1 and S2, respectively (S3 Fig). Furthermore, the presence of quadriradial chromosome formations were not observed, which are present in around 2% of Bloom syndrome cells [33]. Control lymphocytes showed no detectable chromosome breaks.

**Fig 2:**
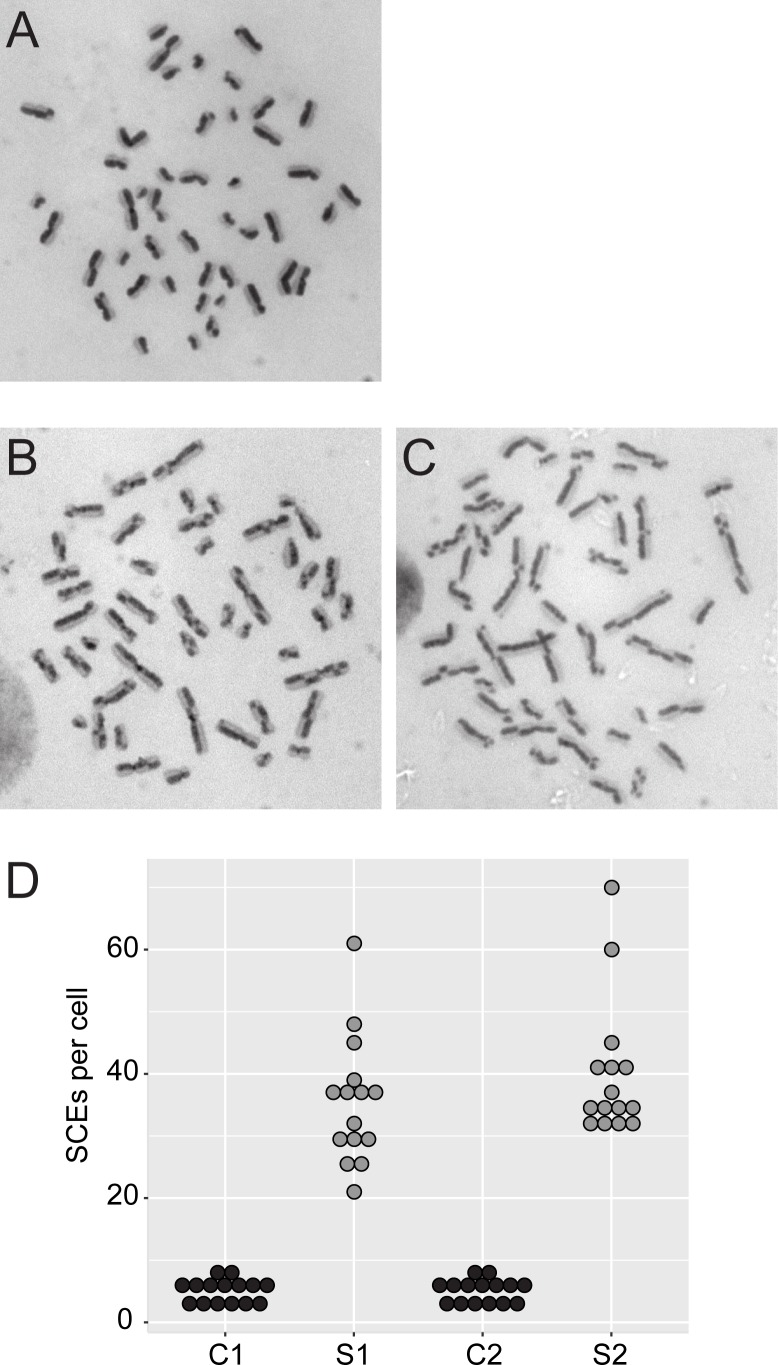
Hyper-recombination in RMI2-deleted individuals. Strand-specific labelling of sister chromatids stain dark and light in control lymphocytes (A) and sib 1 and 2 (B) and (C), respectively. (D) Sister chromatid exchanges were counted from 15 cells per individual. C1 and 2 are sex and age-matched control cells.

To confirm the cytological phenotype of elevated sister chromatid exchange events that is characteristic of Bloom-like syndrome, fresh peripheral blood lymphocytes were prepared for differential sister chromatid staining. Both affected siblings and two sex and age-matched controls were assayed microscopically for sister chromatid exchanges. 15 cells from each individual were examined and showed a mean of 40 and 36 chromatid crossovers for S1 and S2, respectively, compared with a mean of five crossovers for controls (Fig 2).

**Fig 3:**
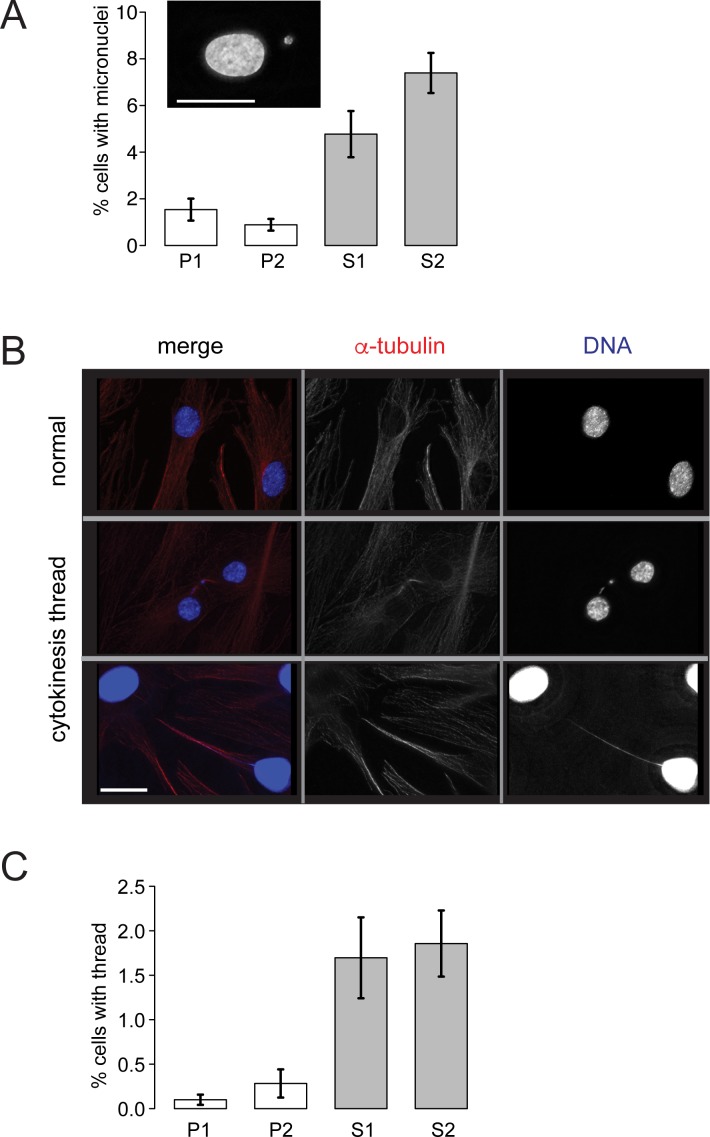
RMI2 suppresses chromosome mis-segregation events. Fibroblasts grown on coverslips were fixed and then stained with DAPI. (A) Scoring of fibroblast cells with micronuclei. At least 3000 cells were scored from each of P1, P2, S1, S2 cells in matched cell passage number from four independent experiments. Example of a cell with a micronucleus is shown in inset, scale bar 15 μm. (B) Representative cells showing DNA bridges connecting interphase cells. Top panel shows normal and the bottom two panels show affected interphase cells with interconnecting DNA threads. Note the bottom cell has had the DAPI/DNA (blue) signal enhanced to visualise the DNA thread. Cells were co-stained with anti-α-tubulin (red). Scale bar 5 μm. (C) Scoring of fibroblast cells with interphase interconnecting DNA threads. At least 3000 cells were scored from each of parental control (P1, P2) and RMI2 deficient siblings (S1, S2) in matched cell passage number from three independent experiments. Error bars represent standard error of the mean.

To examine the extent of chromosome entanglements in mitosis, fibroblast cell lines were established from the siblings and parents. These cell lines enabled a number of cytological analyses to be performed. Fibroblasts were grown on coverslips and then fixed and stained with DAPI. The presence of micronuclei are a useful biomarker for chromosomal breaks and missegregation events [34]. The number of cells containing at least one micronucleus was 4.8% and 7.4%, S1 and S2, respectively (Fig 3A). By contrast, the parents showed 1.5% and 0.89%, for P1 and P2, respectively. This equates to a 5 to 8-fold higher frequency of micronuclei in the siblings fibroblast cells.

**Fig 4:**
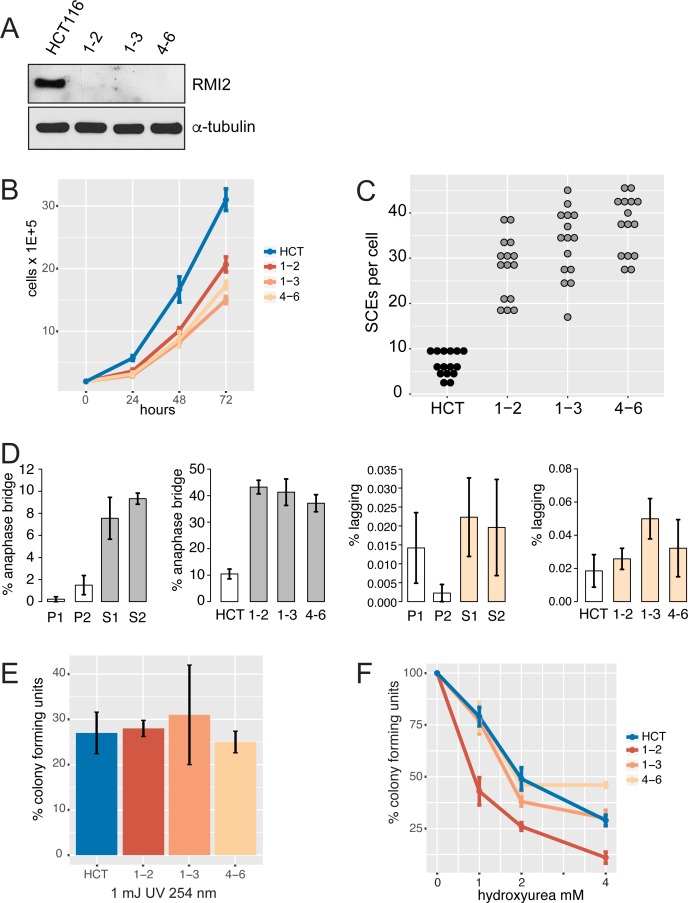
RMI2 cellular defects in knockout cell lines. (A) Immunoblot of HCT-116 wild type and three independent RMI2 null clones (1–2, 1–3 and 4–6) confirms loss of RMI2 protein in gene knockout cells. Equivalent cell extract (40 μg) was loaded in each lane with anti-α-tubulin used as a loading control. (B) Cell proliferation analysis over three days performed in triplicate for each cell line. (C) Sister chromatid exchange analysis on parental and the three RMI2 null clones. Fifteen metaphase cells were analysed for each cell line. (D) Quantification of anaphase bridges and lagging chromosome from parental heterozygote, P1, P2, and homozygous siblings S1 and S2, and in RMI2 wild type and null HCT-116 cells. For fibroblasts (P1, P2, S1, S2) at least 200 anaphase/telophase cells were scored in total for each line from four independent experiments using matched cell passage number. For HCT-116 cells, at least 200 anaphase/telophase cells were scored for each of wild-type HCT-116, and RMI2 null clones 1–2, 1–3 and 4–6 from three independent experiments using matched cell passage number. Error bars represent standard error of the mean. (E) Colony forming and UV sensitivity assays on HCT-116 and null cell lines. The total number of colonies from three independent experiments are normalised against untreated cells. (F) Colony forming and hydroxyurea sensitivity assays on HCT-116 and null cell lines. Experiments were normalised as in the UV-challenge experiment. Error bars represent standard error of the mean.

Other features of mitotic errors were also measured. Chromatin threads or bridges connecting interphase nuclei were 0.10% and 0.28% for P1 and P2, respectively, compared with 1.7% and 1.9%, S1 and S2, respectively (Fig 3B and 3C). Larger masses of chromatin in the form of bulky DNA bridges were 0.22% and 1.5% for P1 and P2, respectively, compared with 7.5% and 9.3% for S1 and S2, respectively (Fig 4D, S6 Fig). Although both siblings share the exact same homozygous deletion spanning *RMI2*, overall S2 was more affected than S1 across several mitotic assays. The differences between S1 and S2 however were not statistically significant. This is consistent with her (S2) more severe clinical presentation and growth defects when compared against her brother (S1).

### Confirmation of RMI2 mitotic phenotype in an independent human cell line

In order to confirm the cytological results observed in the fibroblast cells lines, we chose to create independent isogenic knockout cell lines in the near-diploid human colorectal cell line, HCT-116 using CRISPR/Cas9 gene editing. To minimise off-target mutations we adopted a double-nicking strategy [35]. Two separate guide oligonucleotide pairs were used to generate several candidate *RMI2* knockout cell lines. Two knockout cell lines (1–2 and 1–3) from guide pair 1AB and one cell line (4–6) from guide pair 4AB were used in subsequent functional characterisation. Details of the mutations are provided in (S4 Fig). The three knockout cell lines were confirmed to be null for the RMI2 protein with immuno-blot (Fig 4A). These cell lines provided an independent additional line to support and expand observations from fibroblast patient cells.

The HCT-116 rate of SCEs per cell was 6.6 per cell compared with a combined average of 34 for the three KO clones (Fig 4C and S5A–S5D Fig). This equates to a 5.2-fold increase in the *RMI2* knockout cell lines. Anaphase bridges showed four-fold increase in frequency when compared to wild-type cells. Whereas, lagging chromosome frequency displayed a modest increase over wild-type cells (Fig 4D and S6 Fig). Together, data from HCT-116 replicate findings from our patient fibroblast cell lines, with RMI2 null cells showing increased chromosome bridges compared to controls. In both experiments using patient fibroblast and HCT-116 cells there was no significant increase in cells showing lagging chromosomes, suggesting RMI2 and the BTR complex does not play a role in spindle-kinetochore attachment. DNA content analysis was performed on exponentially growing asynchronous cells from fibroblast and HCT-116 cell lines to determine if there was any polyploidy or aberrant cell cycling. No differences were observed between wild-type and RMI2 null cell lines (S7 Fig).

### RMI2 null cells form smaller and fewer colonies but are not sensitive to UV light or hydroxyurea

We were interested to ascertain whether RMI2 loss affected cell proliferation and UV sensitivity. A previous study in DT40 null cells had shown no effect on cell proliferation rates or sensitivity to DNA damaging chemicals [11], whereas another study using RNAi knockdown in human cells had observed a lower survival rate in cells challenged with MMS [10]. To assess whether the loss of RMI2 had an impact on cell proliferation and colony forming ability, 300 cells were plated onto dishes and grown for six days before being fixed and analysed. The RMI2 null cells showed a 2.4-fold and 8.9-fold decrease over parental wild-type cells for number of colonies and the total area that the colonies occupied per well, respectively (S5E and S5F Fig).

To test whether the RMI2 null cells were sensitive to UV light, 300 cells were plated per well and allowed to recover for one day before being exposed to UV light. The average number of colonies in the RMI2 null cells dropped to 27% of untreated cells, compared with a similar drop of 27% for untreated wild-type cells (Fig 4E). We also challenged the cells with the DNA replication inhibitor, hydroxyurea (HU) at varying doses (Fig 4F). No consistent sensitivity was observed in the knockout cell lines.

### Ultrafine bridges dramatically increase in cells without RMI2

Our study analyzed bulky DNA bridges and found a significant increase due to the absence of RMI2 (Fig 4D, S6 Fig). Another class of bridge that is associated with the BTR complex activity are ultra fine bridges (UFBs) that are finer, thread-like structures not detectable using DAPI. BLM is one of several proteins that co-localise with UFBs in the later stages of mitosis with members of the BTR appearing as a streak between separating chromosomes most commonly during early anaphase [32]. UFBs occur naturally in mitosis and although the precise function of BTR in cells undergoing chromosome segregation is still to be determined, it is thought the complex aids sister chromatid decantation during anaphase [36]. It is presumed that UFBs associate with loci that contain un-replicated DNA or unresolved recombination intermediates that persist into mitosis [17]. Relevant to this study, patients with Bloom syndrome show significantly elevated levels of UFBs as a result of defective BLM [32].

**Fig 5:**
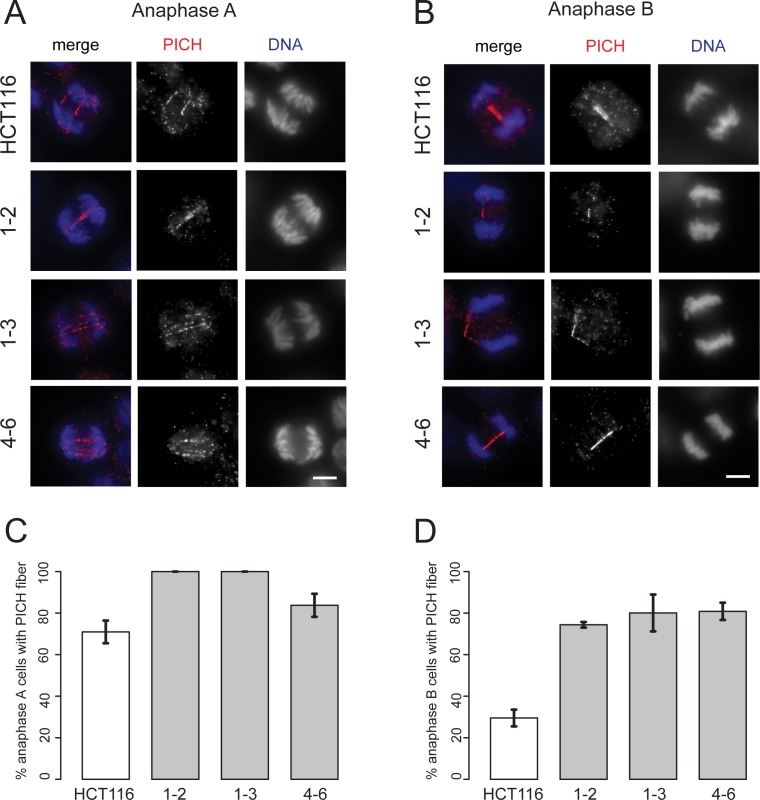
Loss of RMI2 causes significant elevation in UFBs. Representative images and RMI2 wild-type and null HCT-116 cells stained with anti-PICH (red) and DAPI for DNA (blue) for anaphase A (A) and anaphase B (B). Scale bar 5 μm. Quantification of PICH fiber detection in anaphase A cells in (C) and anaphase B (D) from wild-type HCT-116 control and RIM2 null cells (1–2, 1–3, 4–6). Data taken from three independent experiments, with 20 anaphase A and 20 anaphase B cells scored for each HCT-116 cell line (wild-type, 1–2, 1–3, 4–6) per experiment. Error bars represent standard error of the mean.

To test whether RMI2 null cells also showed significant increases in UFBs, HCT-116 wild type and null RMI2 cells were stained with Plk1-interacting checkpoint helicase (PICH) protein (Fig 5), which colocalises with BLM on UFBs during anaphase [31,32]. PICH is considered a useful marker of UFBs as its localisation is independent of the BTR. The results were striking and paralleled analogous scoring in BLM disrupted cells [32]. We found that there was only a slight increase in anaphase A cells displaying PICH fibers between wild-type and RMI2 null HCT-116 clones (Fig 5A and 5C). However, by anaphase B approximately only 30% of wild type cells shows detectable PICH fibers, while the approximately 80% in null HCT-116 RMI2 null cells (Fig 5B and 5D). The data clearly indicate UFBs persist into anaphase B as a result of RMI2 disruption.

### BTR complex impairment on ultra fine anaphase DNA bridges

**Fig 6:**
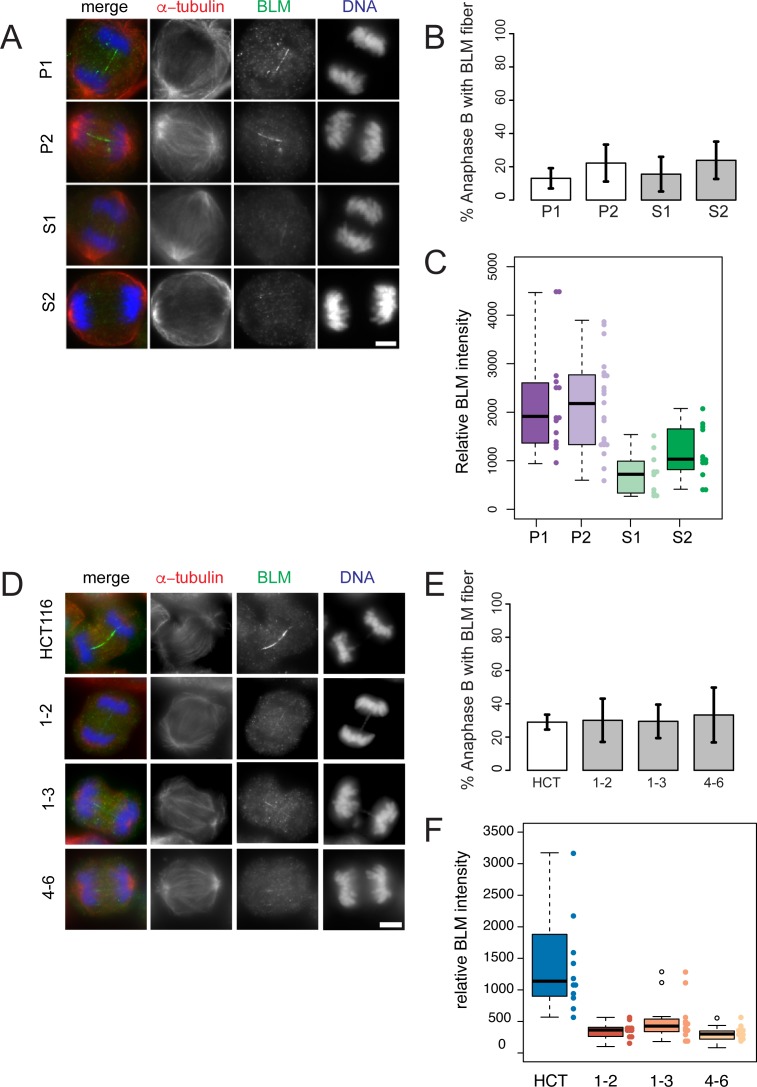
BLM fibers are weaker in anaphase B cells lacking RMI2. Representative anaphase B images of parental heterozygous, P1 and P2, and homozygous siblings S1 and S2, fibroblasts (A) and RMI2 wild-type and null HCT-116 cells (D) stained with anti-BLM (green), anti-α-tubulin (red) and DAPI for DNA (blue). Scale bar 5 μm. For anaphase A analysis see S8 Fig. Quantification of BLM-staining fibers in anaphase B cells in (B) parent (P1, P2) and sibling (S1, S2), and (E) wild-type HCT-116 control and RIM2 null cells (1–2, 1–3, 4–6). Data taken from three independent experiments, with a minimum of 15 anaphase B cells scored for each fibroblast cell line (P1, P2, S1, S2) per experiment and also for each HCT-116 cell line (wild type, 1–2, 1–3, 4–6) per experiment. Error bars represent standard error of the mean. Quantification of BLM fiber intensity on fibroblast cell line (P1, P2, S1, S2) (C) and wild-type HCT-116 and RIM2 null anaphase cells (F). Data for C, F pooled from anaphase A and B cells from two independent experiments.

BTR complex members are a set of several proteins that co-localise with UFBs in the later stages of mitosis. We therefore analysed core components to determine if their localizations were altered in the absence of RMI2. BLM appears as a streak on UFBs between separating chromosomes, with BLM fibers most evident during early anaphase [32]. Although the precise function of BLM in cells undergoing chromosome segregation is still to be determined, it is thought the complex aids sister chromatid decantation during anaphase. Furthermore, it is presumed that UFBs associate with loci that contain un-replicated DNA or unresolved recombination intermediates that persist into mitosis [17], however the precise nature of the DNA is yet to be described. Examination of BLM localisation on anaphase fibroblast cells revealed little difference compared to controls in the prevalence of positively-staining fibers from both affected siblings and RMI2 HCT-116 null cells in anaphase B (Fig 6B and 6E). What was apparent however was the intensity of BLM (using pooled data from anaphase A and B) on the fiber was significantly weaker in RMI2 null cells in both patient and HCT-116 systems compared to controls (Fig 6A, 6C, 6D and 6F). Interestingly, in anaphase A there was small, but statistically insignificant drop in detection of BLM-positive fiber in HCT-116 RMI2 null cells relative to wild-type and an even larger decline in the analogous experiment in using patient fibroblast lines (S8 Fig).

**Fig 7:**
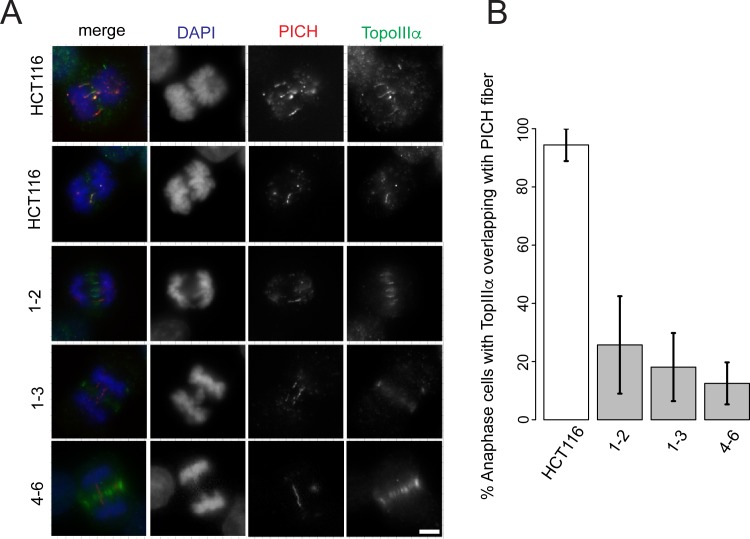
RMI2 is necessary for localization of TopoIIIα to anaphase UFBs. Representative images of RMI2 wild-type and null HCT-116 cells (A) stained with anti-TopoIIIα (green), anti-PICH (red) and DAPI for DNA (blue). Scale bar 5 μm. (B) Quantification of anaphase B HCT-116 wild-type and RMI2 null cells detected with PICH and TopoIIIα. Numbers represent only the pool of anaphase B cells with PICH fibers and whether these demonstrated colocalization with TopoIIIα. The data was taken from three independent experiments, with a minimum of nine anaphase cells per experiment per cell line.

We next examined TopoIIIα localisation onto UFBs in anaphase (Fig 7). We adopted a slightly different approach and measured the amount of anaphase B cells that showed PICH and TopoIIIα colocalisation. Our previous data (Fig 5) showed approximately 30% of anaphase cells had PICH fibers, so we asked the question how many of these PICH fibers show colocalisation with TopoIIIα. The results were very clear. For wild type HCT-116 cells 94% of anaphase B cells with PICH overlapped with TopoIIIα compared 26%, 18% and 13% for RMI2 HCT-116 null clones 1–2, 1–3 and 4–6 respectively. Together the results show RMI2 is necessary for the proper localization of the BTR complex members BLM and TopoIIIα, and provide a mechanistic link why UFBs persist during anaphase B in RMI2 null cells *i*.*e*., due to disruption of BTR subunits in anaphase.

**Fig 8:**
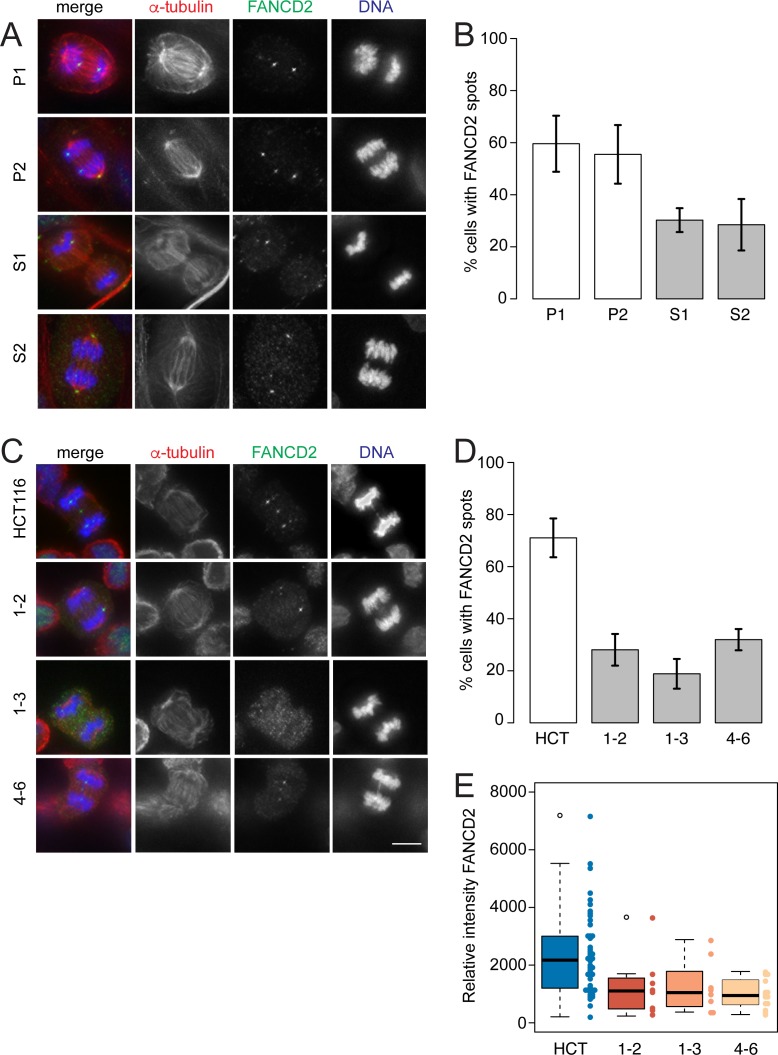
FANCD2 sister foci are reduced in RMI2 deficient cells. Representative images of parental heterozygous, P1 and P2, and homozygous S1 and S2, fibroblasts (A) and RMI2 wild-type and null HCT-116 cells (C) stained with anti-FANCD2 (green), anti-α-tubulin (red) and DAPI for DNA (blue). Scale bar 5 μm. Note the FANCD2 signal in a DAPI negative area (presumably a UFB), connecting bulky bridged DNA in HCT-116 wild-type cells. Occasionally the FANCD2 antibody recognised centrosomes (see image P2, S2), which was discounted in all scoring. Quantification of anaphase/telophase cells detected with FANCD2 sister foci in (B) parent (P1, P2) and sibling (S1, S2) and (D) HCT-116 wild-type and RMI2 null cells. Data taken from three independent experiments, with 13–30 anaphases scored for each fibroblast cell line (P1, P2, S1, S2) per experiment and 30 anaphases scored for HCT-116 cells (wild-type, 1–2, 1–3, 4–6) per experiment. Error bars represent standard error of the mean. (E) Quantification of FANCD2 spot intensity on HCT-116 wild-type and RIM2 null cells in anaphase/telophase cells. Data pooled from two independent experiments.

The Fanconi anaemia (FANC) complex is needed for the repair of DNA ICLs generated during DNA replication [23]. Subunits of the FANC and BTR complexes interact together forming a super-complex known as BRAFT [22]. Furthermore, the FANCD2/FANCI subunits forms foci at regions of replication stress such as common fragile sites that anchor the BLM-staining fibers between segregating sister chromatids [17]. The FANCD2/FANCI foci on separating chromatids are visible from anaphase through to telophase [29]. We also noticed, FANCD2 can occasionally appears as a fiber across separating chromatids, reminiscent of the BLM and PICH (S9 Fig). We have examined the localisation of FANCD2 on UFBs in the family’s fibroblasts and the HCT-116 RMI2 null cells. Both cell types show a decrease in the frequency of anaphase to telophase cells containing FANCD2 foci on sister chromatids (Fig 8 and S9 Fig). Additionally, the intensity signals of the FANCD2 foci on the HCT-116 RMI2 null cells show a decrease in signal ranging from 1.9- to 2.4-fold. Taken together, these results suggest the stability of the BRAFT super-complex encompassing BLM and FANCD2 subunits is compromised through loss of RMI2.

## Discussion

### Loss of RMI2 produces a Bloomoid phenotype

We have identified a homozygous deletion of the *RMI2* gene that results in a Bloom-like phenotype from a consanguineous kindred. The two affected siblings exhibit a variable phenotype with some overlapping features of Bloom syndrome. Sibling, S2, presented with growth deficiency and gastro-esophageal reflux, traits commonly found in Bloom syndrome children. Curiously, these indicators were absent in sibling S1 (Table 1). It is too early to tell whether homozygous deletion of *RMI2* is associated with elevated risk of cancer in late childhood or adulthood. Consistent with the clinical presentation, our cell biology analyses also indicated sibling S2 was slightly more affected with mitotic assays for chromosome bridges in mitosis, bridges persisting between interphase nuclei and micronuclei all elevated compared to sibling S1. The striking feature consistent with a Bloom syndrome phenotype, is both children display café-au-lait macules (Fig 1A). These dermatological findings are often associated with childhood cancer syndromes [37]. Loss of RMI2 should therefore be added to the differential diagnosis of children presenting with multiple café-au-lait macules.

Cytogenetic investigation into genome instability showed a higher rate of SCEs and chromatid breaks (Fig 2). BLM null individuals have a 10-fold elevation in the rate of SCEs when compared to wild-type cells [3]. By contrast, we have observed a slightly lower rate at seven- to eight-fold above wild-type. This is consistent with a similar decrease when SCE rates are compared between BLM and RMI2 knockout chicken DT40 cells [11]. These data suggest that the BLM helicase displays partial activity in dissolving catenated DNA in the absence of RMI2. Indeed, in vitro addition of RMI2 to BLM-TopoIIIα-RMI1 caused a statistically significant increase in the rate and overall level of dissolution of radiolabelled double Holliday junction substrates [10]. The same study found the BLM-TopoIIIα-RMI1 complex alone still possesses significant dissolution activity; suggesting RMI2 enhances but is not essential to the enzymatic capability of the BTR complex. However, we note our in vivo analyses show removal of RMI2 has a profound affect on the stability of BLM on UFBs that likely represent a range of replication intermediates. Understanding the DNA structure of UFBs remains an important task that will provide insight into why and how UFB-associated proteins act.

The notion that BLM-TopoIIIα-RMI1 alone can still dissolve double Holliday junctions is consistent with the observation that BLM patients show a more noticeable clinical presentation compared to RMI2 affected individuals. Additionally, BLM has activities that are independent of RMI2. For instance, it is known BLM stimulates the resection activity of human exonuclease 1 [38]. It is therefore also likely that with the increase in DNA analysis capabilities and also clinical awareness that further RMI1 and also RMI2 affected individuals will be identified in the population.

The failure to dissolve catenated DNA in the affected siblings is the main trigger for downstream mitotic errors such as DNA anaphase bridges and micronuclei (Fig 3 and Fig 4). These perturbations in mitosis are thought to have an impact on the cell proliferation rate. We have investigated whether there was any link between mitotic errors and growth rates in the affected siblings but no consistent association could be found. Homozygous knockout of the *RMI2* gene in HCT-116 cells showed a noticeable slowing down in cell proliferation and the ability to form colonies from single cells (Fig 4B and S5E and S5F Fig). This is in contrast to the chicken DT40 RMI2 knockout cell lines that did not display any reduction in cellular growth rate, although colony forming assays were not performed [11]. Correspondingly, one of the affected siblings showed prenatal and postnatal growth deficiency (Table 1). The variability in cell proliferation rates and impact on development is most likely to be dependent on genetic background. Furthermore, the growth deficiency phenotype observed in sibling S2 may be due to a co-existing disorder associated with the parents’ consanguinity. RMI2 mouse knockout studies will hopefully shed some light on these differences between model systems.

DNA repair disorders are commonly associated with sensitivity to DNA-damaging agents such as chemical mutagens or short wave radiation such as UV light. Bloom syndrome affected individuals are mildly sensitive to sunlight where they display sun-sensitive lesions on exposed areas such as the face [1]. However, there are conflicting reports in the literature whether BLM null cells are sensitive to UV light in vitro [4,39,40]. The affected siblings did not show any signs of sun-sensitive skin lesions on exposed areas. HCT-116 RMI2 null cells also did not show any consistent reduction in the number or size of colonies after being exposed to short-wave UV light or hydroxyurea when compared to parental wild-type cells (Fig 4). Similar results were observed in chicken DT40 RMI2 null cells when challenged with DNA-damaging chemicals such as cisplatin or methyl methanesulfonate [11]. Taken together, these data support that the BLM helicase can perform some of its DNA repair functions without the participation of RMI2.

### RMI2 suppresses defects during the later stages of mitosis

Earlier experiments on the knockdown of RMI2 from vertebrate cells had not examined its role during chromosome segregation. Examination of RMI2 null cells in fibroblasts and HCT-116 lines has shown hallmarks of mitotic errors in the form of DNA bridges and micronuclei (Fig 3 and Fig 4). Were these chromosome entanglements due to the lack of the BTR complex localising to UFBs? We have shown both BLM (Fig 6) and TopoIIIα (Fig 7) localisation is disrupted when RMI2 is removed. Our data however show that BLM still can localise to UFBs, albeit at a significantly lower intensity. Together, this evidence supports our hypothesis that the BTR is functionally impaired during mitosis without RMI2.

Further evidence of this partial activity is illustrated with localisation experiments of FANCD2 in RMI2 null cells. Like BLM, FANCD2 sister chromatid foci are reduced in frequency and intensity, suggesting that BTR instability impacts upon important DNA repair complexes such as FANC (Fig 8). This is not without precedent as it is known that BLM co-immunoprecipitates with FANCD2 in human cells [41], and mechanistically FANCD2 and the BTR complex cooperate to restart stalled replication forks [28,42]. These studies suggest a physical and mechanistic interplay between BTR and FANCD2 in S phase under replication stress and the dependencies seemingly persist through to M phase. Curiously ours (Fig 8 and S9 Fig) and another study [29] observed FANCD2 coated UFBs. The nature of FANCD2 UFBs has not been fully explored, but it is possible they exist as backup or additional activity in resolving catenated DNA structures during anaphase.

The BLM Bloom syndrome gene was first identified over 20 years ago. Our report shows that this is the first clinical description of individuals with Bloomoid features of non-BLM subunit. Although producing a clinical presentation similar to Bloom syndrome, the hallmark features are not as severe. Independent studies using cell lines derived from homozygous affected siblings and also HCT-116 cells deleted of *RMI2* both show overlapping defects with marked increase in DNA bridges during the later stages of cell division. Our data show removing RMI2 affects the stability of interacting partners in the BRAFT super complex with BLM and FANCD2 reduced on chromosomes during chromosome segregation. Significantly, those cells without RMI2 that displayed BLM fibers in anaphase showed a marked drop in signal intensity, suggesting RMI2 stabilises or activates the complex. Whether there is any residual activity of BLM and how the overall subunit composition and architecture of the BTR and BRAFT complexes is affected is not yet clear. These will be important questions for future studies. Our current study suggest both at the patient and cell biology level the effects are not as severe as lacking BLM altogether.

## Materials and Methods

### Subjects

Family members were recruited to this study with the approval of the Hospital Research Ethics Committee at the Royal Children’s Hospital, Melbourne, Australia, ethics approval number, 28097. Written consent for the affected individuals was provided by their parents.

### Genomic microarray

Genomic DNAs were isolated and purified from leukocytes using the NucleoSpin Tissue genomic DNA extraction kit (Machery-Nagel, Germany). DNA samples were processed by the Illumina Infinium method using the HumanCytoSNP—12 v2.1 (Illumina, San Diego, CA, USA) microarray platform and analysed using KaryoStudio v1.4 software (Illumina). Confirmation of the null deletions and cascade testing of the parents was performed using Affymetrix CytoScan 750K array using the manufacture’s protocols and analysed using Chromosome Analysis Suite vCytoB-N1.2.2.271 (Affymetrix, Thermo Fisher Scientific).

### Cell lines

Fibroblast and HCT-116 cell lines were cultured in BME and RPMI, respectively. Media were supplemented with 10% FBS and penicillin/streptomycin.

### Breakpoint PCR and sequencing

Primers were designed next to the closest positive microarray probe on either side of the breakpoints. The following oligonucleotides (IDT), RM-delf (5’—CCTACTCCTCCTGCCCTTTTC—3’) and RM-delr (5’—CCTGCCTCTTTACCTGGAGTG—3’) were used in a long-range PCR amplification reaction using Phusion Hot Start II (Thermo Fisher Scientific) with the following conditions; 98°C 2 min (1 cycle), 98°C 30 sec, 61°C 30 sec, 72°C 3 min (40 cycles), 72°C 10 min (1 cycle). PCR products were A-tailed with AmpliTaq Gold DNA polymerase (Thermo Fisher Scientific) 72°C 10 min, and cloned into pGEM-T Easy (Promega) using standard methods. The plasmid insert was Sanger sequenced using primer walking at the Australian Genome Research Facility, Melbourne, Australia.

### Sister chromatid exchange assay

Fresh blood cells were incubated for three to four days in RPMI 1640 media/10% FBS with 20 μg/ml phytohaemagglutin. BrdU (Sigma-Aldrich) was added to a final concentration of 10 μg/ml for 30 hours followed by 0.1 mg/ml colcemid (Thermo Fischer Scientific) treatment for 45 mins before standard metaphase chromosome harvest. HCT-116 cell lines were treated for 29 hours with 10 μg/ml BrdU, followed by 0.1 mg/ml colcemid for 1.5 hours. Phosphate buffer pH 6.8 was added to cover the dried slides to a depth of 2 mm. Slides were then placed in a biosafety cabinet and were exposed to UV light at a distance of 30 cm for 45 min. The slides were briefly rinsed in dH_2_O and added to prewarmed 2 x SSC at 65°C for 30 min, followed by another rinse in dH_2_O and stained in Leishman’s stain (Sigma-Aldrich).

### UV treatment

RMI2-null HCT-116 clones were seeded onto 6-well dishes at 300 cells in three ml of media per well in triplicate for each cell line. The next day the cells were exposed to either 2 mJ UV (254 nm) or mock treatment using a GS Gene Linker UV Chamber (Bio-Rad). Cells were then grown for six days and then rinsed in PBS, fixed in ice-cold methanol and stained in crystal violet solution. The 6-well dishes were imaged and colonies of at least 0.032 mm^2^ were counted using ImageJ v2.0.0.

### Immunoblot analyses

Cell extracts preparation for immunoblotting was performed as described before [43]. In brief, cells were collected and washed once with cold PBS. The pellets were resuspended in RIPA buffer with fresh prepared EDTA-free protease inhibitor (Roche) and incubated on ice for 15 min and then sonicated. Protein concentration were determined using the Quick Start Broadford Protein Assay (Bio-Rad). 40 μg of total protein extract from each of the samples was run on 10% SDS PAGE gels (Bio-Rad). The following antibodies were used for immunoblot detection, rabbit polyclonal anti-RMI2 (1:1000) (Abcam), mouse monoclonal anti-α-tubulin antibodies (1:1000)(Sigma-Aldrich), swine anti-rabbit IgG-HRP (1:10,000)(Dako) and rabbit anti-mouse IgG-HRP (1:10,000)(Dako). ECL immuno-blotting substrate (Pierce) was used according to the manufacturer’s instructions.

### Immunofluorescence

Fibroblasts or HCT-116 cells were seeded onto gelatinised 22 mm x 22 mm glass coverslips in 6-well trays. After at least 24 hours, media was removed and cells were rinsed in PBS. For immunofluorescence cells were fixed with 4% paraformaldehyde for 10 minutes, permeabilised with 0.3% Triton X-100 and blocked with 3% BSA in PBS. Cells were stained with rabbit polyclonal anti-BLM (1:500)(Abcam), rabbit monoclonal anti-FANCD2 (1:500)(Abcam), mouse-monoclonal anti-PICH (Millipore, 1:200), rabbit polyclonal anti-TopoIIIα (kind gift from the Hickson laboratory, University of Copenhagen, 1:200) and mouse monoclonal anti-α-tubulin antibodies (Sigma, 1:500). Secondary antibodies were donkey anti-rabbit Alexa Fluor 488 (1:1000)(Invitrogen) and goat anti-mouse Alexa Fluor 594 (1:1000)(Invitrogen). Cells were mounted with VectaShield containing DAPI (Vector Laboratories).

### Microscopy and image analysis

For sister chromatid exchange and breakage analyses, methanol-acetic acid fixed preparations were imaged using a Zeiss Axioplan 2 microscope with a 100× objective lens. Images were analysed using AxioVision 4.7 (Zeiss). For FANCD2 images taken by DeltaVision, 36 sections (0.2 μm per section) images were taken. Images were deconvolved, and projected in 2D using SoftWoRx 4.1. Percentage of cells with symmetrical FANCD2 spots were scored and plotted. Obvious symmetrical FANCD2 spots intensity were further measured using the polygon function of SoftWoRx 4.1. For BLM fibers scoring and intensity measurements, images were captured using Zeiss Axio Imager M1 microscope and processed by AxioVision 4.7 (Zeiss). Percentage of cells with BLM fibers were scored and plotted. Line profiles across the fibers in the cells were analysed using ImageJ as described before [44].

### CRISPR/Cas9 knockout

Two independent nicking CRISPR/Cas9 guide pairs were designed using the CRISPR design tool at crispr.mit.edu. Both pairs targeted the coding sequence of exon 2. The following target sites for nicking pair #1, Guide A minus (5'—TCCCACATACTTTCATGGATGGG– 3'), Guide B plus (5'—TGGAGGTAGAAGATTTACACAGG—3') and #4 Guide A minus (5'—ATCTTCACAGCCTGCAGGCAGGG—3'), Guide B plus (5'—TCCCATCCATGAAAGTATGTGGG– 3'). Annealed oligonucleotides were cloned into the pSpCas9n(BB)-2A-GFP (PX461) vector (Addgene plasmid ID: 48140) [35]. HCT-116 cells were transfected in 6-well trays with Lipofectamine 3000 (Thermo Fisher Scientific) using the supplier's protocol. Two days after transfection, GFP-positive single cells were flow sorted into 96-well trays. Genomic DNA from clones was extracted using standard methods followed by PCR amplification screening across the CRISPR target site using the following oligonucleotides; RM-mf (5'—GATGGTGATGGGAGTGGTTC—3') RM-mr (5'–TCCTACATCCGGACTCCTTG—3'). PCR products were cloned into pGEM-T Easy (Promega) and Sanger sequenced at the Australian Genome Research Facility to confirm the presence of a knockout mutation. Three clones with knockout alleles at the DNA and protein levels were chosen for functional characterisation.

### Flow cytometry

DNA content analysis was performed as previously described [43] and analysed using FACSCalibur and Cell Quest (Becton Dickinson).

### Statistical analyses

Box plots were generated using beeswarm R package (https://cran.r-project.org/web/packages/beeswarm/index.html). Histograms were generated using Hmisc package (http://cran.r-project.org/web/packages/Hmisc/index.html). Statistical analyses were conducted using Student’s t test (unpaired).

## Supporting Information

S1 Fig SNP-array results across the deleted region. Each sib, (A) S1 and (B) S2, display a small, 80-kb homozygous interstitial deletion (orange vertical bar) spanning the RMI2 gene on chromosome 16. Note the lack of SNP heterozygosity across the region, exhibited by blue dots.
(PDF)

S2 Fig Sequence characteristics of the deletion junction. Two Alu elements on the telomere (tel) and centromere (cen) sides of the deleted region show evidence of a non-allelic recombination event. (A) Sequence alignment of the two elements reveals a high level of homology and the position of the recombination event (arrow). Sequence chromatogram across the deletion junction (arrow).
(PDF)

S3 Fig Elevated chromatid breaks in RMI2-deleted individuals. Block stained metaphase chromosomes from control lymphocytes show intact chromosomes (A). Siblings 1 (B) and 2 (C) contain chromatid breaks, shown by arrow.
(PDF)

S4 Fig RMI2 CRISPR-Cas9 mutation sequence sites of null clones. CRISPR-Cas9 induced RMI2 mutation regions were PCR-amplified, cloned and sequenced. Deletion or insertion region is shown against the reference genome for each nickase pair. Guide pairs are shown in blue with the protospacer adjacent motif (PAM) site shown in red.
(PDF)

S5 Fig Sister chromatid exchange and colony forming analyses on RMI2 null clones. (A–D) Differential chromatid staining on representative metaphase cells from HCT-116 and RMI2 null clones, 1–2, 1–3 and 4–6. (E, F) Colony forming assays on HCT-116 and RMI2 null cell lines displayed as numbers of colonies and total area occupied in a 6-well tray (arbitrary units).
(PDF)

S6 Fig Examples of anaphase bridges and chromosome laggards in RMI2 null cells. Representative images of dividing fibroblasts showing bridges and lagging chromosomes. Cells were co-stained with anti-α-tubulin (red) and DAPI to visualise DNA (blue). Scale bar 5 μm.
(PDF)

S7 Fig DNA content analysis on fibroblasts and knockout cell lines Exponentially growing cells were measured for DNA content using flow cytometry. (PDF)

S8 Fig Analysis of BLM fibers in Anaphase A wild-type and RMI2 null cells. Representative anaphase A images of parental heterozygous, P1 and P2, and homozygous siblings S1 and S2, fibroblasts (A) and RMI2 wild-type and null HCT-116 cells (B) stained with anti-BLM (green), anti-α-tubulin (red) and DAPI for DNA (blue). Scale bar 5 μm. Quantification of detection of BLM fibers in anaphase A cells in (C) parent (P1, P2) and sibling (S1, S2), and (D) wild-type HCT-116 control and RIM2 null cells (1–2, 1–3, 4–6). Data taken from three independent experiments, with a minimum of 15 anaphases A cells scored for each fibroblast cell line (P1, P2, S1, S2) per experiment and also for each HCT-116 cell line (wild type, 1–2, 1–3, 4–6) per experiment. Error bars represent standard error of the mean.
(PDF)

S9 Fig FANCD2 occasionally localises to UFBs during anaphase. Examples of FANCD2 localisation to sister chromatid foci in parental fibroblast cells, P2. A fine thread of FANCD2 signal can be seen in an anaphase cell of sibling S2. Fibroblast cells stained with anti-FANCD2 (green), anti-α-tubulin (red) and DAPI for DNA (blue). Scale bar 5 μm.
(PDF)
