# Supplementary material for: Loss of RMI2 Increases Genome Instability and Causes a Bloom-Like Syndrome
Source: PLoS Genet. 2016 Dec 15;12(12):e1006483. doi: 10.1371/journal.pgen.1006483 (PMC5157948; doi:10.1371/journal.pgen.1006483)
Supplement: S2 Fig — Two Alu elements on the telomere (tel) and centromere (cen) sides of the deleted region show evidence of a non-allelic recombination event. (A) Sequence alignment of the two elements reveals a high level of homology and the position of the recombination event (arrow). Sequence chromatogram across the deletion junction (arrow). (PDF) [file pgen.1006483.s002.pdf]

A

```
tel    1     GGCCAGGCGCGGTGGCTCACACCTGTAATCCCAGCACTTTGGGAGGCCGAGGCGGTCGGA  60
             ||||||| || ||||||||  ||| |||||||| |||||||||||||| |||| |  |||
cen    1     GGCCAGGTGCAGTGGCTCATGCCTCTAATCCCAACACTTTGGGAGGCCAAGGCAGGTGGA  60

tel    61    TCACG--AGGTCAGGAGATCGAAACCATCCTGGCTAACACGGTGAAACCCTGTCTCTACT  118
             ||||   |||||||||| | || |||| |||||| |||| ||   |||||||||||||||
cen    61    TCACCTAAGGTCAGGAGTTTGAGACCAGCCTGGCCAACATGGCAGAACCCTGTCTCTACT  120

tel    119   AAAAATACAAAAAATTAGCCGGGCGTGGTGGCGGGCGCCC--GTAGTCCCAGCTACTAGG  176
             ||||||||||||||||||| ||| | | ||||  |||| |  ||||||||||||||||||
cen    121   AAAAATACAAAAAATTAGCTGGGTGGGATGGCACGCGCACCTGTAGTCCCAGCTACTAGG  180

tel    177   GAGGCTGAGGCAGGAGAATGGCATGAATCCGGGAGGCGGAGCTTGCAGTGAGCCAAGATT  236
             |||||||||||||||||||| | ||||  | ||| | | || || |||||||||||||||
cen    181   GAGGCTGAGGCAGGAGAATGACTTGAACTCAGGAAGTGAAGGTTTCAGTGAGCCAAGATT  240

tel    237   GCTCCACTGCACTCCAGCCTGGGTGGC-AGAGACAGACCTTGTCTCAAAATAATAACAAT  295
             || |||||| |||| ||||||||   | ||||  | ||    |||||||| || || || 
cen    241   GCACCACTGTACTCTAGCCTGGGCAACAAGAGCAAAACTCCATCTCAAAAAAAAAAAAAA  300

tel    296   AATAATAA  303
             || || ||
cen    301   AAAAAAAA  308
```

green - retained
black - deleted

B

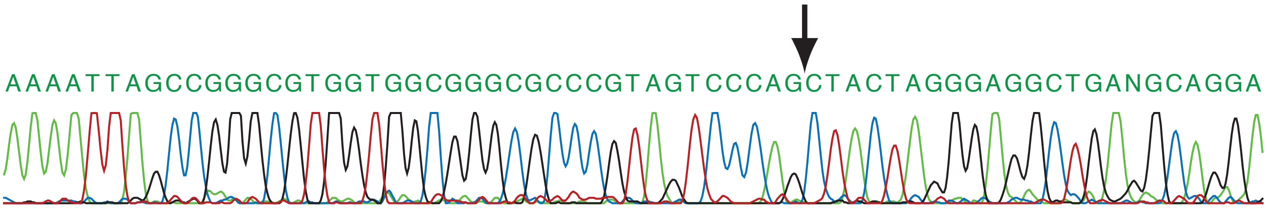

Supplementary Figure 2
