# Supplementary figures and images for: Loss of RMI2 Increases Genome Instability and Causes a Bloom-Like Syndrome
Source: PLoS Genet. 2016 Dec 15;12(12):e1006483. doi: 10.1371/journal.pgen.1006483 (PMC5157948; doi:10.1371/journal.pgen.1006483)

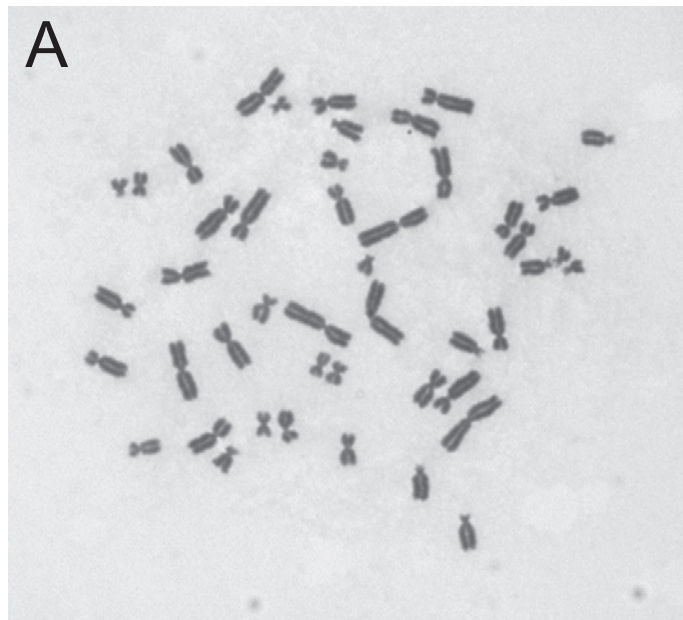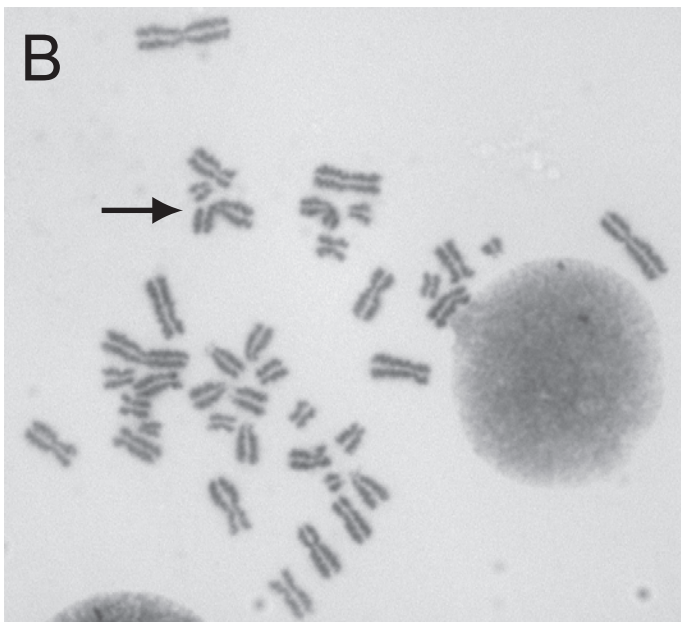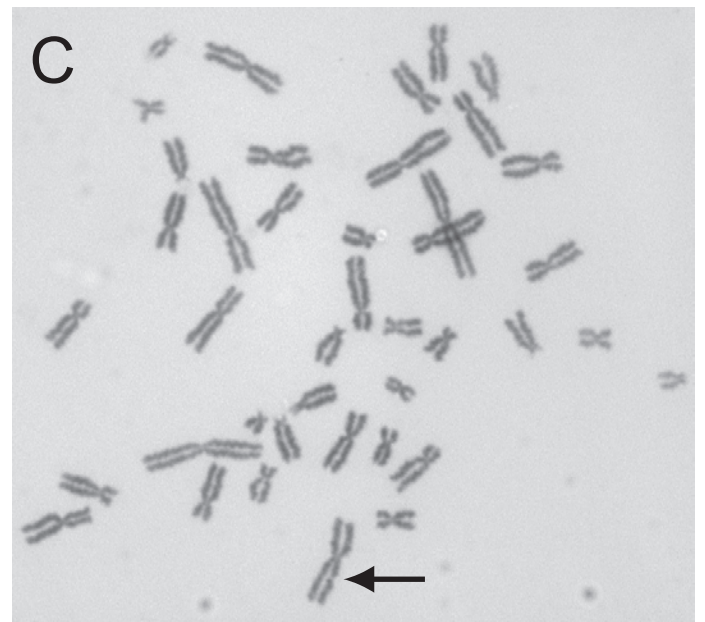

Supplementary Figure 3

Supplement: S3 Fig — Block stained metaphase chromosomes from control lymphocytes show intact chromosomes (A). Siblings 1 (B) and 2 (C) contain chromatid breaks, shown by arrow. (PDF) [file pgen.1006483.s003.pdf]

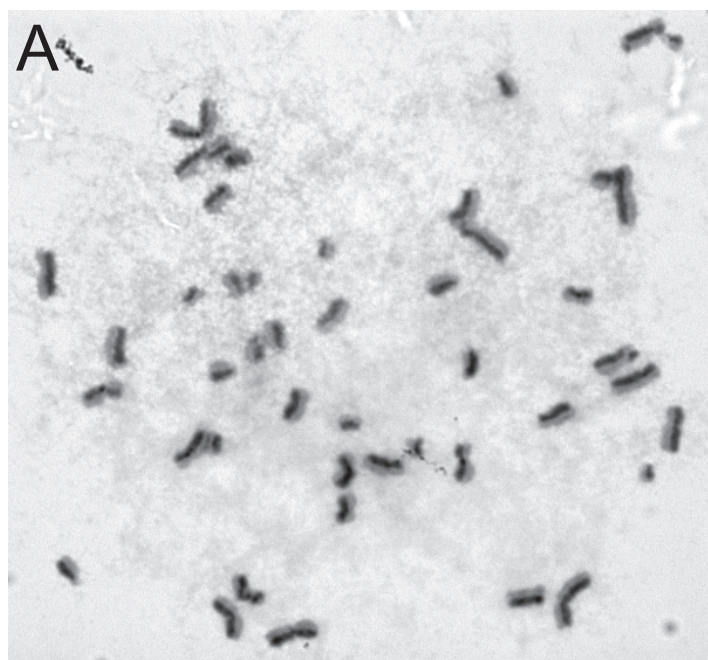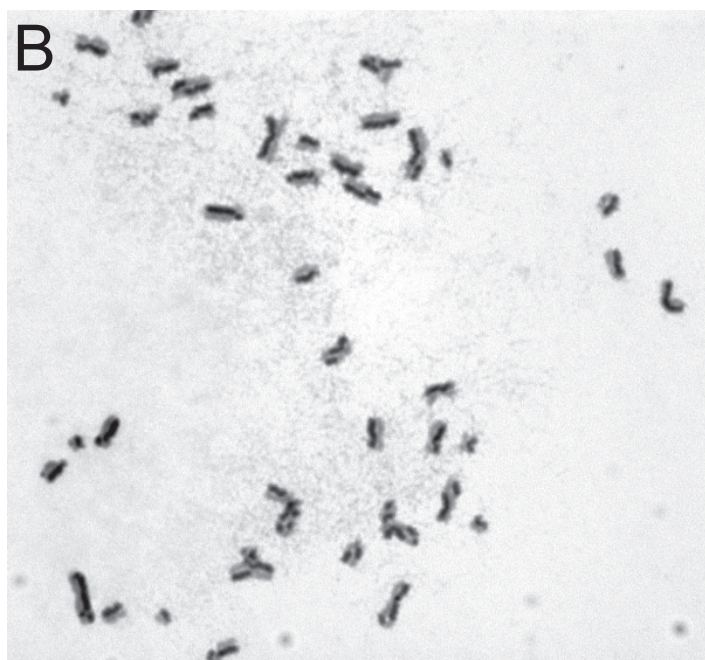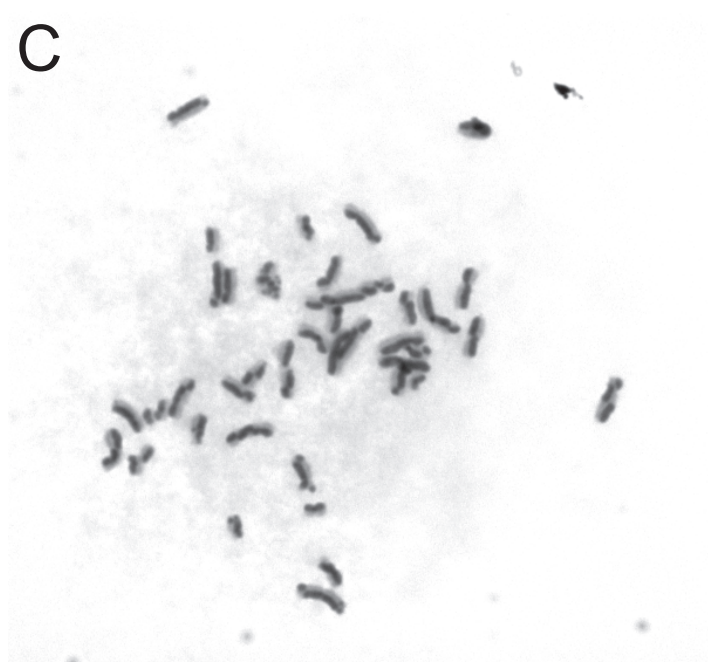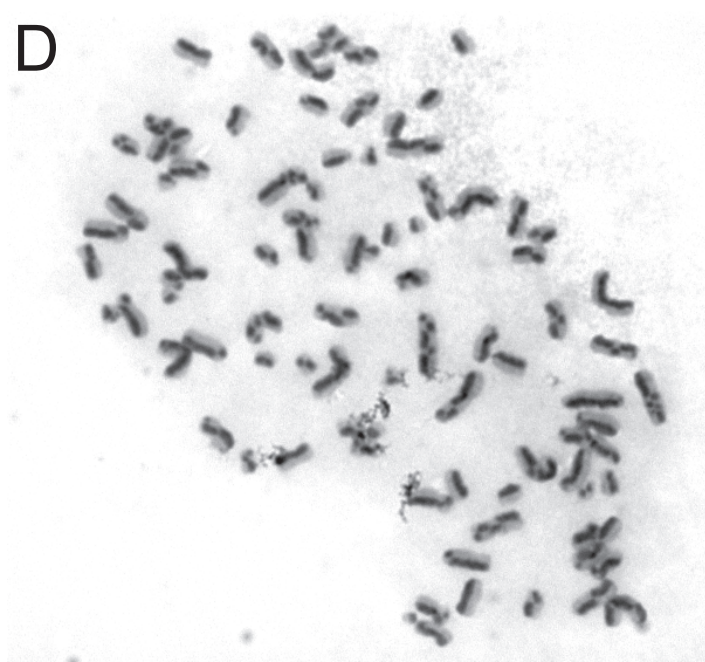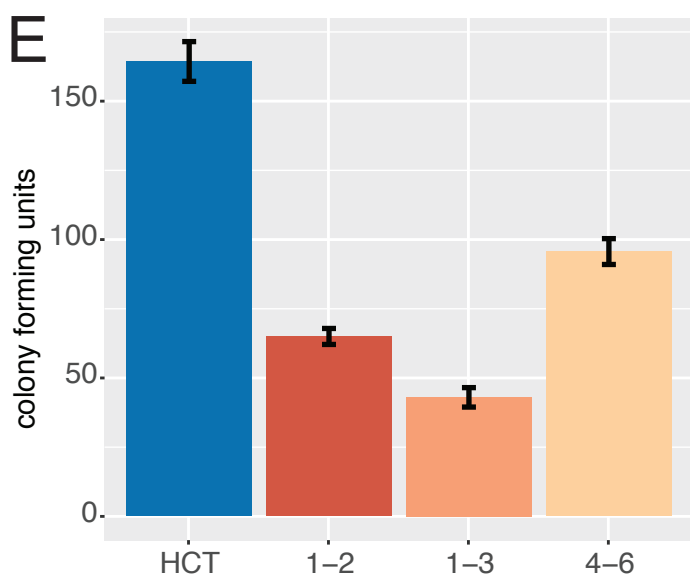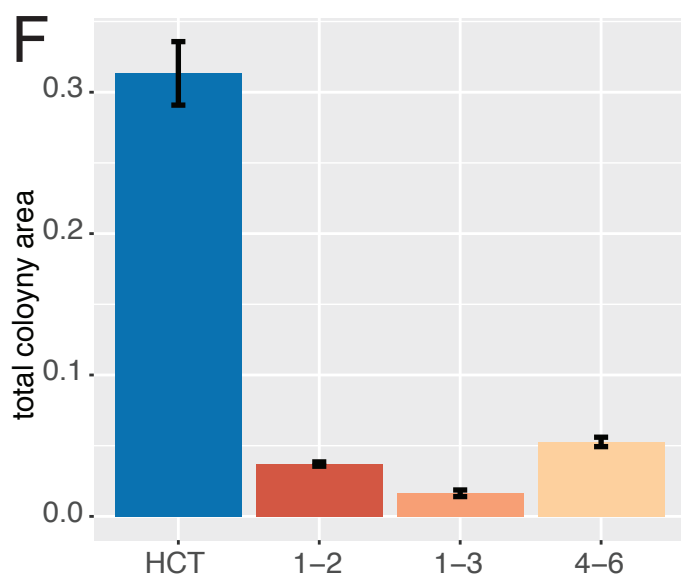

Supplementary Figure 5

Supplement: S5 Fig — (A–D) Differential chromatid staining on representative metaphase cells from HCT-116 and RMI2 null clones, 1–2, 1–3 and 4–6. (E, F) Colony forming assays on HCT-116 and RMI2 null cell lines displayed as numbers of colonies and total area occupied in a 6-well tray (arbitrary units). (PDF) [file pgen.1006483.s005.pdf]

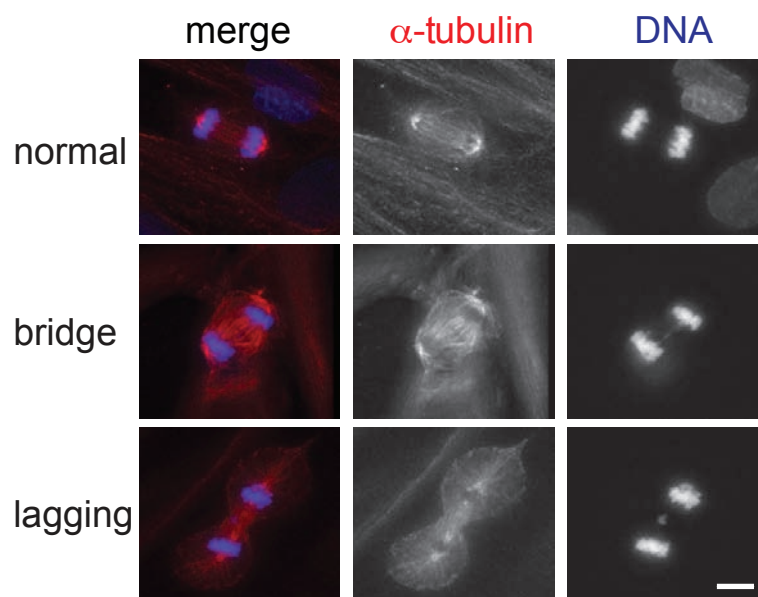

Supplementary Figure 6

Supplement: S6 Fig — Representative images of dividing fibroblasts showing bridges and lagging chromosomes. Cells were co-stained with anti-α-tubulin (red) and DAPI to visualise DNA (blue). Scale bar 5 μm. (PDF) [file pgen.1006483.s006.pdf]

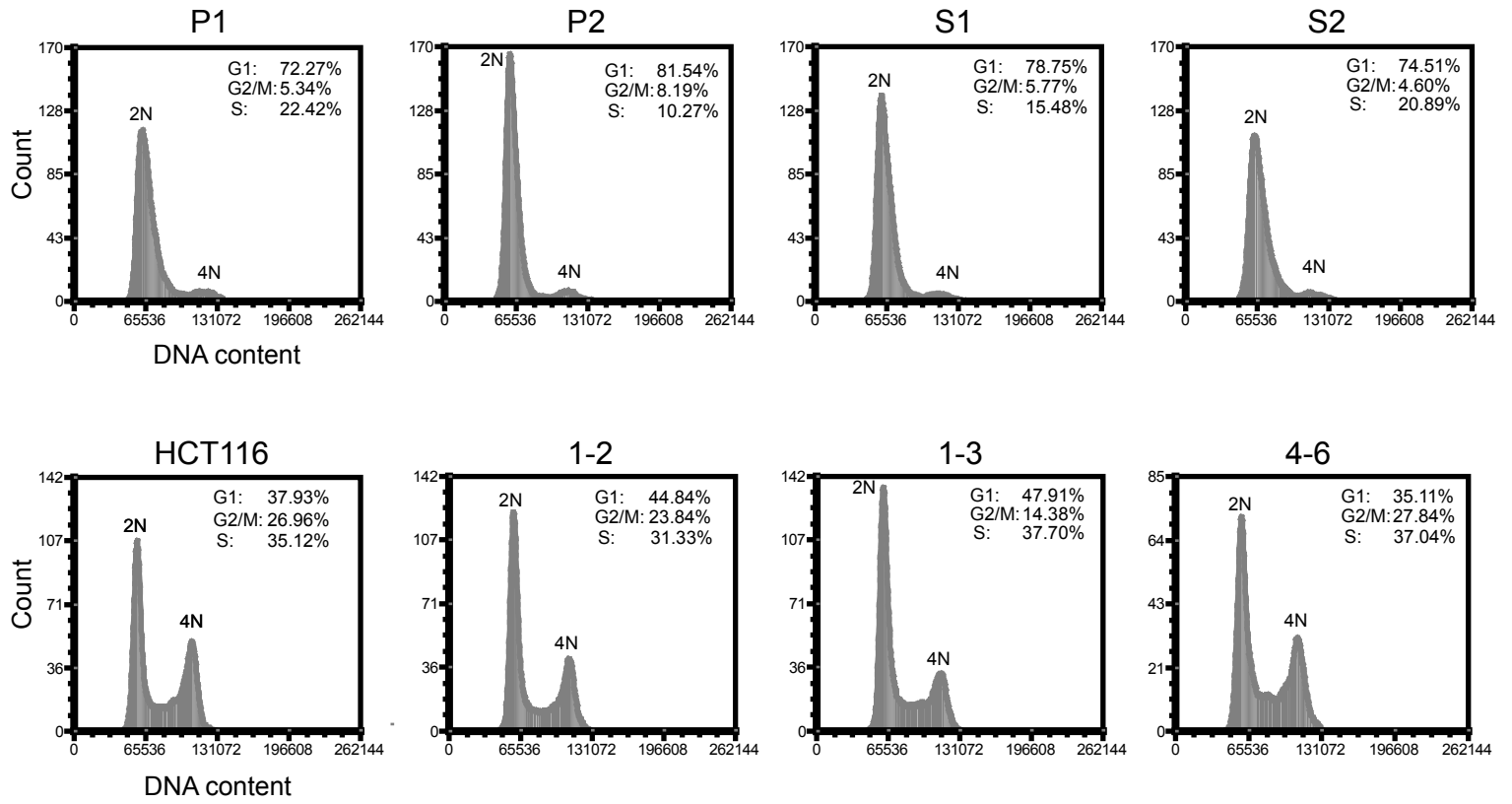

Supplementary Figure 7

Supplement: S7 Fig — (PDF) [file pgen.1006483.s007.pdf]

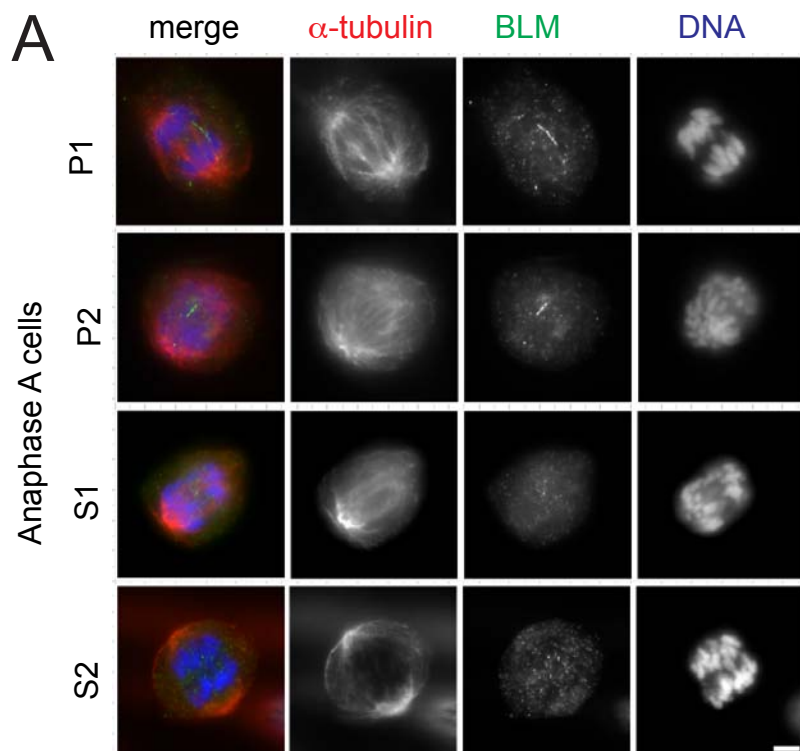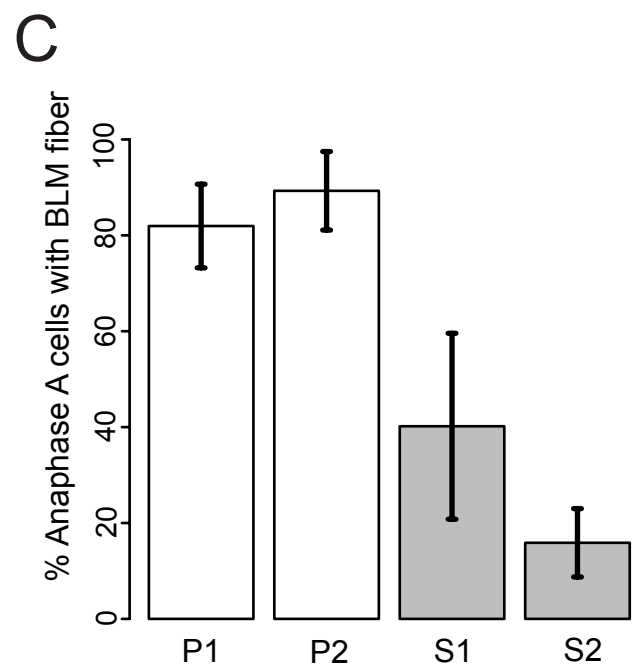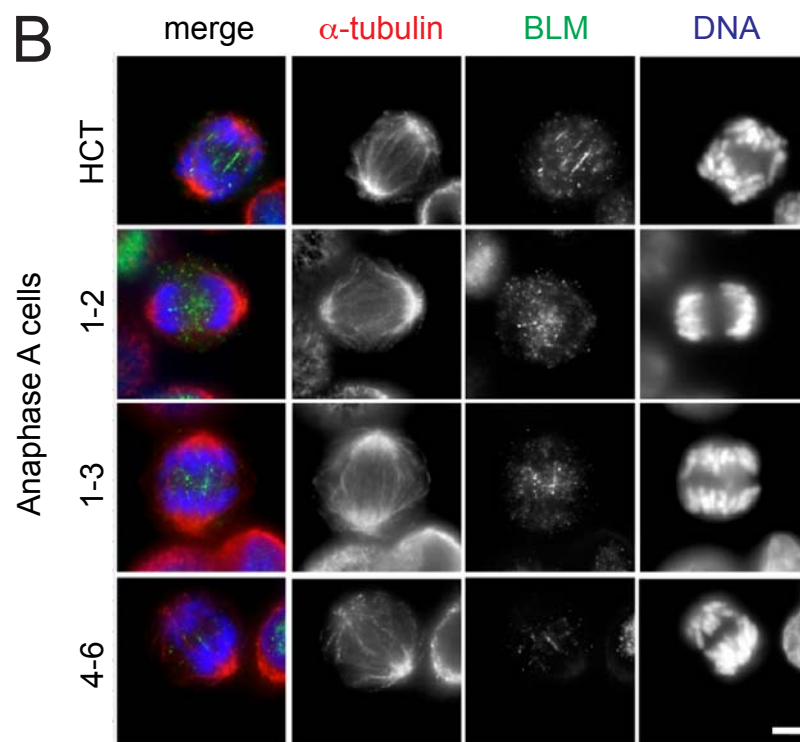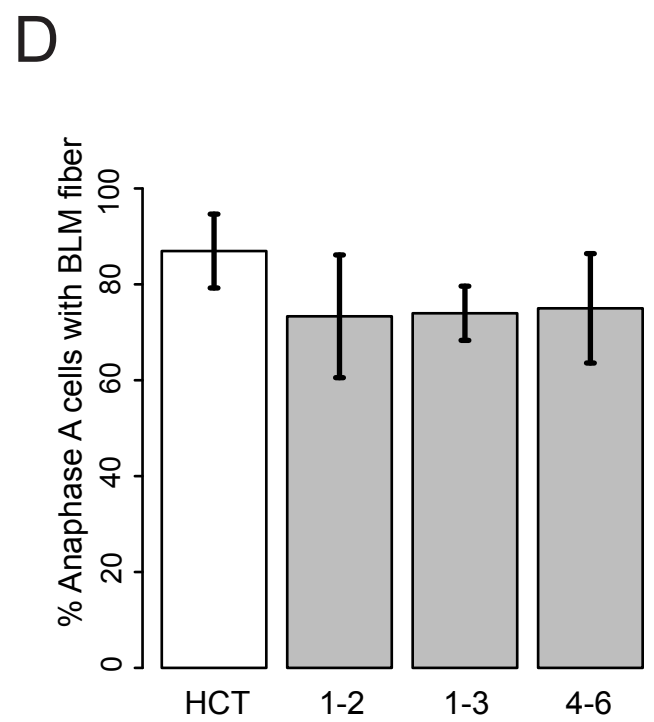

Supplementary Figure 8

Supplement: S8 Fig — Representative anaphase A images of parental heterozygous, P1 and P2, and homozygous siblings S1 and S2, fibroblasts (A) and RMI2 wild-type and null HCT-116 cells (B) stained with anti-BLM (green), anti-α-tubulin (red) and DAPI for DNA (blue). Scale bar 5 μm. Quantification of detection of BLM fibers in anaphase A cells in (C) parent (P1, P2) and sibling (S1, S2), and (D) wild-type HCT-116 control and RIM2 null cells (1–2, 1–3, 4–6). Data taken from three independent experiments, with a minimum of 15 anaphases A cells scored for each fibroblast cell line (P1, P2, S1, S2) per experiment and also for each HCT-116 cell line (wild type, 1–2, 1–3, 4–6) per experiment. Error bars represent standard error of the mean. (PDF) [file pgen.1006483.s008.pdf]

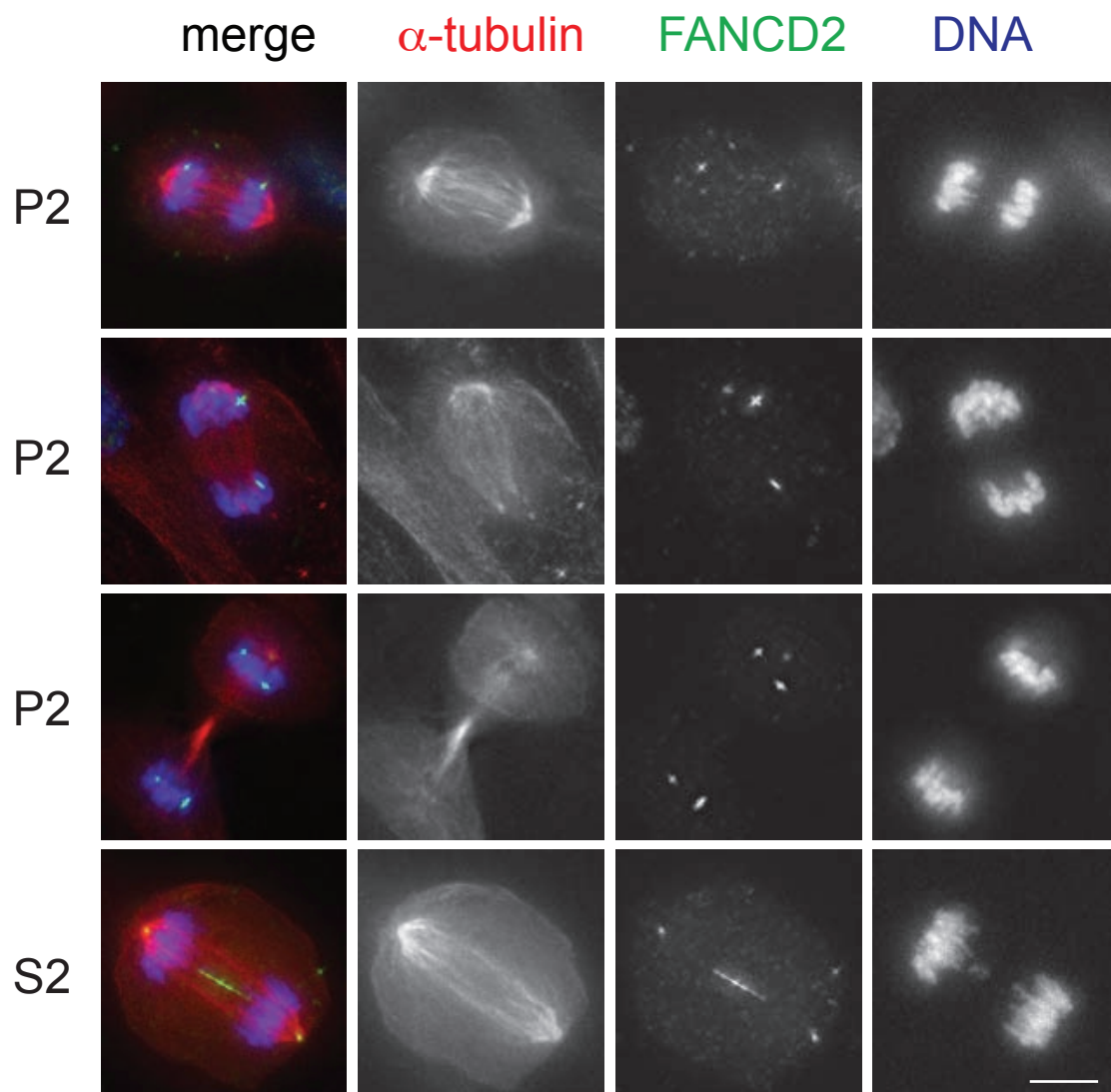

Supplementary Figure 9

Supplement: S9 Fig — Examples of FANCD2 localisation to sister chromatid foci in parental fibroblast cells, P2. A fine thread of FANCD2 signal can be seen in an anaphase cell of sibling S2. Fibroblast cells stained with anti-FANCD2 (green), anti-α-tubulin (red) and DAPI for DNA (blue). Scale bar 5 μm. (PDF) [file pgen.1006483.s009.pdf]
