# Supplementary material for: Loss of RMI2 Increases Genome Instability and Causes a Bloom-Like Syndrome
Source: PLoS Genet. 2016 Dec 15;12(12):e1006483. doi: 10.1371/journal.pgen.1006483 (PMC5157948; doi:10.1371/journal.pgen.1006483)
Supplement: S4 Fig — CRISPR-Cas9 induced RMI2 mutation regions were PCR-amplified, cloned and sequenced. Deletion or insertion region is shown against the reference genome for each nickase pair. Guide pairs are shown in blue with the protospacer adjacent motif (PAM) site shown in red. (PDF) [file pgen.1006483.s004.pdf]

1AB guide pair

```
ref + CCTTTCTGATAATCCCATCCATGAAAGTATGTGGGAACTGGAGGTAGAAGATTTACACAGGAATATTCCTTAGAGTATGTTGGAAC
                   GGGTAGGTACTTTCATACACCCT
                                            TGGAGGTAGAAGATTTACACAGG
ref - GGAAAGACTATTAGGGTAGGTACTTTCATACACCCTTGACCTCCATCTTCTAAATGTGTCCTTATAAGGAATCTCATACAACCTTG

1-2   CCTTTCTGATAATCCCATCCATGAAAGTATGTGGGAACTGG-- 14-bp del -CACAGGAATATTCCTTAGAGTATGTTGGAAC

1-2   CCTTT-- 14-bp del -CATGAAAGTATGTGGGAACTGGAGGTAGAAGATTTACACAGGAATATTCCTTAGAGTATGTTGGAAC

1-3   CCTTTCTGATAATCCCATCCATGAAAGTATGT----------------- 48-bp del –-------------------TGGAAC
```

4AB guide pair

```
ref + CTGCAGCCCTGAGCCCTGCCTGCAGGCTGTGAAGATGACAGACCTTTCTGATAATCCCATCCATGAAAGTATGTGGGAA
                  GGGACGGACGTCCGACACTTCTA
                                                            TCCCATCCATGAAAGTATGTGGG
ref - GACGTCGGGACTCGGGACGGACGTCCGACACTTCTACTGTCTGGAAAGACTATTAGGGTAGGTACTTTCATACACCCTT

4-6   CTGCAGCCCTGAGCCCTG---------------------- 55-bp del ----------------------TGGGAA

4-6   CTGCAGCCCTGAGCCCTG------------- 95-bp ins + 50-bp del --------------GTATGTGGGAA
```

1-2
14 bp deletion in coding sequence (cds)
c.413_426del

14 bp deletion in cds
c.378_391del

1-3
only one PCR product detectable on gel
48 bp deletion spanning stop
c.402_*5del

4-6
55 bp deletion in cds
c.347_401del

50 bp deletion in cds and a 95 bp insertion
c.348_397delins chr8:33126352-33126446

Supplementary Figure 4
