# Supplementary material for: Loss of RMI2 Increases Genome Instability and Causes a Bloom-Like Syndrome
Source: PLoS Genet. 2016 Dec 15;12(12):e1006483. doi: 10.1371/journal.pgen.1006483 (PMC5157948; doi:10.1371/journal.pgen.1006483)
Supplement: S1 Fig — Each sib, (A) S1 and (B) S2, display a small, 80-kb homozygous interstitial deletion (orange vertical bar) spanning the RMI2 gene on chromosome 16. Note the lack of SNP heterozygosity across the region, exhibited by blue dots. (PDF) [file pgen.1006483.s001.pdf]

A

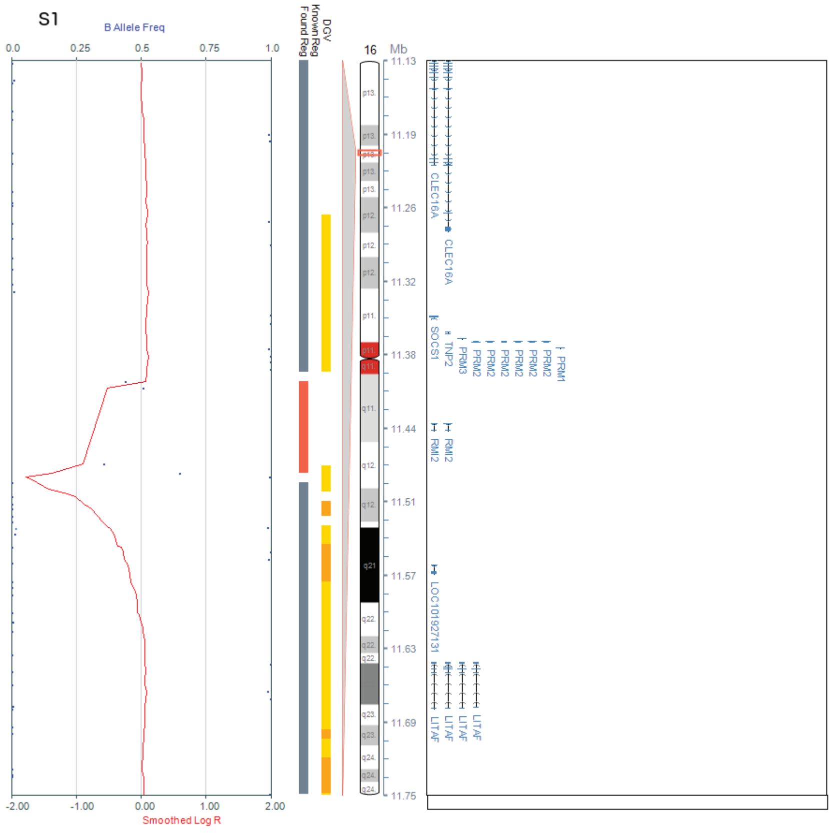

B

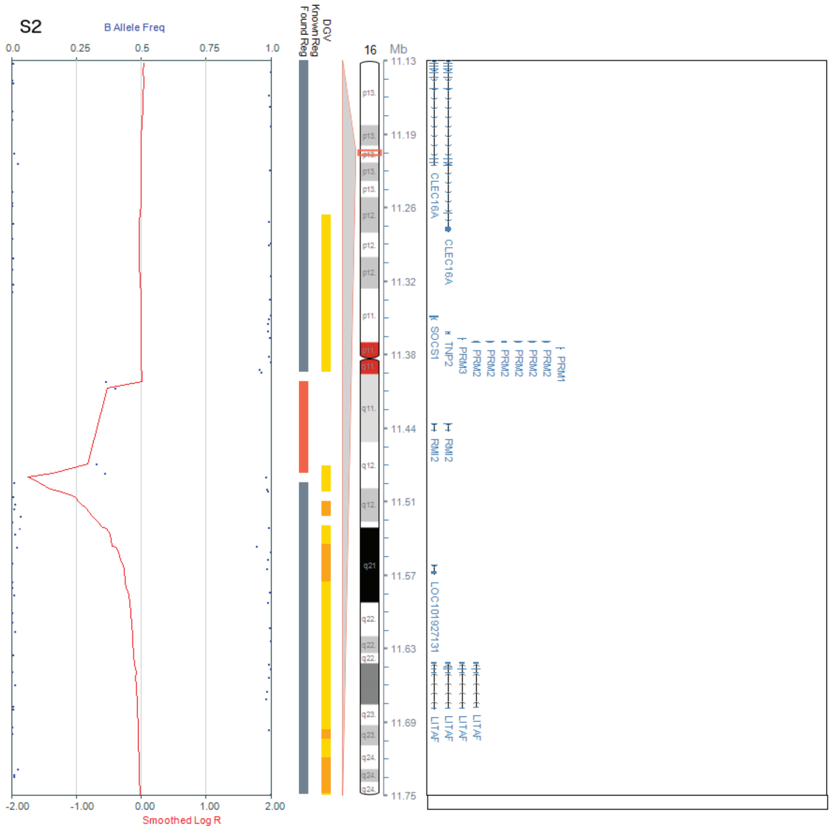

Supplementary Figure 1
